# Incentive Mechanism for Privacy-Preserving Collaborative Routing Using Secure Multi-Party Computation and Blockchain

**DOI:** 10.3390/s24020542

**Published:** 2024-01-15

**Authors:** Chaojie Wang, Srinivas Peeta

**Affiliations:** 1 School of Civil and Environmental Engineering, Georgia Institute of Technology, Atlanta, GA 30332, USA; chaojie.wang@gatech.edu; 2 H. Milton Stewart School of Industrial and Systems Engineering, Georgia Institute of Technology, Atlanta, GA 30332, USA

**Keywords:** incentive mechanism, secure multi-party computation, blockchain, privacy, collaborative routing

## Abstract

Traffic congestion results from the spatio-temporal imbalance of demand and supply. With the advances in connected technologies, incentive mechanisms for collaborative routing have the potential to provide behavior-consistent solutions to traffic congestion. However, such mechanisms raise privacy concerns due to their information-sharing and execution-validation procedures. This study leverages secure Multi-party Computation (MPC) and blockchain technologies to propose a privacy-preserving incentive mechanism for collaborative routing in a vehicle-to-everything (V2X) context, which consists of a collaborative routing scheme and a route validation scheme. In the collaborative routing scheme, sensitive information is shared through an off-chain MPC protocol for route updating and incentive computation. The incentives are then temporarily frozen in a series of cascading multi-signature wallets in case vehicles behave dishonestly or roadside units (RSUs) are hacked. The route validation scheme requires vehicles to create position proofs at checkpoints along their selected routes with the assistance of witness vehicles using an off-chain threshold signature protocol. RSUs will validate the position proofs, store them on the blockchain, and unfreeze the associated incentives. The privacy and security analysis illustrates the scheme’s efficacy. Numerical studies reveal that the proposed incentive mechanism with tuned parameters is both efficient and implementable.

## 1. Introduction

Traffic congestion occurs as a result of an imbalance in demand and supply on a spatio-temporal scale. The well-known Braess’ Paradox demonstrates that relying solely on supply side solutions that focus on increasing the capacity of existing infrastructure without regard for traveler behavior may have a negligible or even detrimental effect on the performance of traffic networks. Demand-side strategies employ behavioral interventions to encourage shifts in travel mode, travel routes, and departure times [1,2]. Of these, en route re-routing is the most challenging problem due to the inherent dynamics and randomness. Over the last few decades, effective behavioral intervention strategies have been developed, such as toll and incentive mechanisms, using model-based [3,4,5,6,7] or model-free [8,9,10] approaches to influence travelers’ en route behavior and thereby alleviate traffic congestion. However, these intervention strategies frequently overlook individual-level heterogeneity, rely on multiple user classes to reflect distinct behavioral patterns, and suffer from computational tractability issues associated with centralized computation.

Emerging connected technologies enable information sharing among vehicles and infrastructure through vehicle-to-everything (V2X) communications in a connected traffic environment [11,12,13,14,15], facilitating more informed collaborations among connected vehicles (CVs) in the re-routing process [16,17,18]. Li et al. [19] proposed a routing method facilitating the navigation through passing time windows in a connected vehicle environment; this has informed the direction of our study. Additionally, Li et al. [20] developed a self-evolving routing method, which introduces a novel formulation in the spatial domain that resolves the mismatch between routing and planning found in conventional studies. This spatial domain-based planning method represents a key contribution to the field of cooperative routing. Nevertheless, individual heterogeneities are still not captured in these studies.

Wang et al. [21] proposed a novel incentive mechanism for collaborative routing in a connected and autonomous vehicular environment, which leverages individual heterogeneity in the route preferences to enhance the system performance while ensuring user satisfaction. A decentralized computational framework was developed to enable efficient network-level deployment. On the other hand, individual heterogeneity necessitates that each passenger discloses their personal preferences (e.g., value of time) as model inputs, which can represent sensitive information that can be exploited. Also, as collaborative routing reveals model outputs, travelers’ updated routes, and incentives to other travelers, it raises privacy issues. Moreover, validation of execution (i.e., validating whether each vehicle travels on the route selected in the incentive mechanism) can cause a substantial computation burden and result in more privacy leaks if traditional centralized methods like sharing GPS traces are used. Further, such privacy risks may act as a barrier to participation in the system for societally vulnerable groups (e.g., sharing a low value of time may indicate a low income level), further impairing mobility equity.

Blockchain has been gaining enormous attention since the whitepaper on Bitcoin [22]. A blockchain is a decentralized ledger with tamper resistance ensured by cryptography. The popularity of blockchain is not limited to cryptocurrencies. Due to its decentralized nature, it has demonstrated its promise in Internet of Things and vehicular ad hoc networks [23,24,25]. However, currently, a blockchain is mainly used to record and share public information such as traffic incidents [26] rather than sensitive personal information in transportation applications. In other studies [27], a blockchain is also used to store virtual credits (which quantify the level of trust that users can place in certain users based on their historical behavior). However, route preferences and historical travel routes are sensitive information that can be potentially leaked even if anonymous identities are used in blockchains. As for the anonymity paradox of Bitcoin, though every Bitcoin account is anonymous, identity information can still be leaked through transaction pattern analysis because every transaction is transparent. The historical travel routes associated with an account can be used to deduce that user’s travel pattern and potentially leak their real-world identity. Therefore, on-chain computation alone does not enable a privacy-preserving incentive mechanism for collaborative routing.

Secure multi-party computation (MPC) is a subfield of cryptography which allows a group to jointly compute a function without disclosing any participants’ private inputs. A few studies [28,29] leverage MPC in intelligent transportation systems to address privacy concerns. However, none of them address its potential for collaborative routing.

To address privacy concerns in collaborative routing for CVs, this study first proposes a privacy-preserving incentive mechanism based on the decentralized mechanism developed in our previous work [21]. Specifically, a collaborative routing scheme is developed to enable travelers to update routes and compute associated incentives following an MPC protocol without disclosing their value of time. Then, a blockchain-based route validation scheme is proposed to securely validate travelers’ whereabouts at checkpoints along selected routes with the assistance of nearby vehicles (i.e., witness vehicles) while allowing them to conceal their trajectories in the blockchain. Combining on-chain and off-chain cryptographic protocols, the proposed incentive mechanism protects sensitive personal information throughout peer vehicle collaborations and prevents malicious parties from conducting pattern-analysis attacks on the blockchain, ensuring that the entire collaborative routing process adheres to a high standard of privacy.

It is worth noting that the privacy concerns mentioned earlier are not specifically associated with the collaborative routing strategy presented in [21]. Rather, they stem from the inherent nature of personalization. To effectively account for the diverse heterogeneities of individual users, personalization necessitates the incorporation of users’ unique characteristics and preferences to generate tailored outputs that cater to their specific interests. Consequently, while [21] primarily addresses the computational efficiency of enabling personalization within the routing context, the current study emphasizes the implementation of structured privacy protection techniques to mitigate the privacy challenges associated with personalization.

The rest of the paper is organized as follows. Section 2 provides an overview of the proposed incentive mechanism. Section 3 presents detailed protocols of the mechanism. Section 4 discusses numerical studies. Section 5 concludes the paper by summarizing contributions and future directions.

## 2. Mechanism Overview

**Figure 1:**
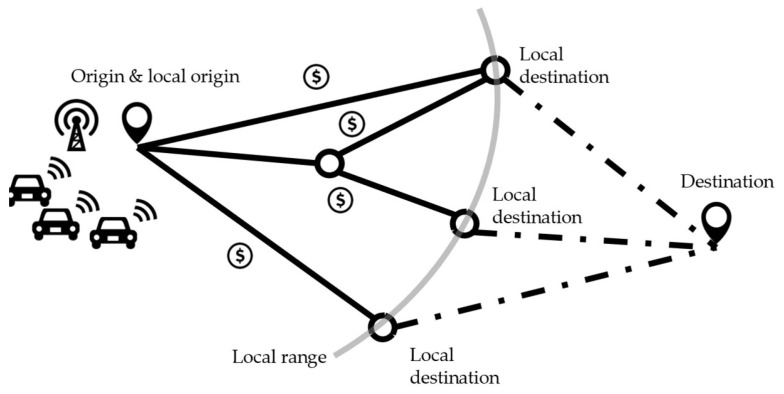
Nudging re-routing behavior with incentives for a local system-optimal assignment (the solid lines and circles denote the road links and nodes within the local range; the dashed lines are the remaining routes from the local destinations to the destination; and dollar signs represent the incentives on the four local routes in the figure).

This study’s problem context is similar to that of our prior study [21]. As depicted in Figure 1, the major objective is to encourage vehicles to reroute collaboratively during their trips in order to improve system performance (specifically, reducing total travel time in this study). When assigning vehicles, the system performance evaluation is based on vehicles’ estimated travel times. Given the difficulty of precisely estimating long-term travel times in the real world, the method will be executed repeatedly throughout the whole horizon of interest. In each iteration, vehicles reroute based on precisely predicted travel times inside the local range (shown by the gray line in Figure 1) and approximations of travel times outside the local range. The decentralized incentive system in [21] follows a hierarchical architecture. Vehicles with the same local origin–destination (OD) pairs are grouped together (the temporary destinations within the defined local range in [21]). First, a route flow assignment model finds the optimal route flows for all vehicle groups in each iteration (with the same local OD pairs). Then, using these optimal flows and the value of time for each vehicle as inputs, a vehicle assignment model assigns vehicles to various routes within each vehicle group. Then, an envy-free procedure produces incentives for every vehicle in the group to ensure participation willingness and behavioral honesty. Notably, in [21], the participation willingness did not account for the potential disadvantages associated with individuals’ privacy concerns about sharing sensitive data during the process. Similarly, whereas behavioral honesty implies that users’ utilities are maximized when they reveal their real value of time, the utility functions did not account for the negative consequences of disclosing the value of time.

For instance, there are four local routes for the group of vehicles in Figure 1. At the end of the current iteration, the vehicles will be provided with four option bundles (incentives calculated by the mechanism are bonded to the corresponding routes). Choosing a certain route determines the bonded amount of incentives. The incentives are designed such that all vehicles picking the bundles that optimize their individual utilities generate a local system-optimal assignment [21]. However, these benefits come with a price in terms of privacy. The vehicle route assignment and incentive calculation depend on vehicles sharing their values of time. And there seems to be no way to prevent incentive scams in which a vehicle claims to choose the option with the highest incentive but later travels on the route with the shortest travel time.

**Figure 2:**
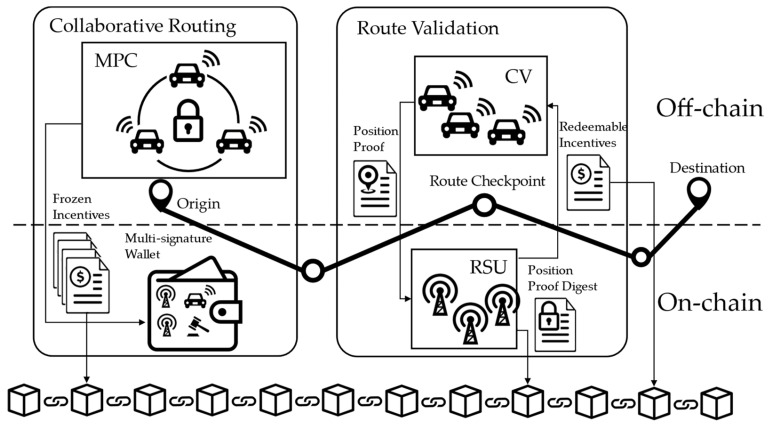
Conceptual structure of the privacy-preserving incentive mechanism for collaborative routing.

Figure 2 depicts the conceptual structure of the privacy-preserving incentive mechanism for collaborative routing, which consists of a secure collaborative routing scheme at the origin (or local origin), and a route validation scheme at the checkpoints along the selected route. Both schemes employ on-chain and off-chain operations (depending on whether they need logging information on the blockchain for the record), ensuring that just the bare minimum amount of data are safely stored on the blockchain (against pattern-analysis attacks). The collaborative routing scheme leverages MPC to securely execute the protocols defined by the vehicle route assignment model and incentive model (steps 2 and 3 in the hierarchical framework) in [21]. Users will not receive the incentives corresponding to the routes they choose at the origin. Instead, the incentives will be temporarily frozen, which means they will be sent to a series of multi-signature wallets/accounts from the traffic operator’s account. The incentives in a multi-signature wallet require *m*-out-of-*n* signatures to become redeemable, where m is the minimum number of signatures required, n is the total number of account holders of the wallet, and both m and n are determined when the wallet is created. Apart from the vehicles receiving these incentives, the account holders of a multi-signature wallet also include one or two verifiers and a mediator. The multi-signature wallets are designed in a cascading manner, such that the frozen incentives can only become redeemable when the user passes the route validation checkpoints along the selected route in order.

When the vehicle is around a checkpoint on the selected route, instead of using GPS information (which can be easily forged by malicious vehicles), it needs to follow the proposed route validation scheme to generate a position proof with the assistance of witness vehicles (nearby vehicles willing to sign on the position proof) and send it to roadside units (RSUs) to verify. The RSUs in the network will verify the proof independently and reach a consensus following the practical byzantine fault tolerance (PBFT) algorithm. If the position proof is valid, a digest of the proof (which is a fixed-size representation of the contents of the proof) will be included in the current block of a consortium blockchain. Meanwhile, the verifier at the checkpoint will sign two multi-signature transactions: one makes the frozen incentives associated with the current checkpoint redeemable for the user; the other one is for the frozen incentives associated with the next checkpoint, which will remain frozen for the time being and requires one more signature to be redeemable. The user does not have to redeem the redeemable incentives (by signing their own signature on the multi-signature transactions and sending them out) at the time he/she receives them. Note that the transactions will be included in the consortium blockchain only when the incentives are redeemed. Therefore, users can hide their trajectories on the blockchain by redeeming the incentives from the checkpoints from multiple trips in arbitrary order.

Leveraging the tamper resistance of the on-chain information while keeping the sensitive information off-chain, the proposed incentive mechanism eliminates both direct privacy leaks (e.g., sharing the value of time in computation) and indirect privacy exposure (e.g., historical trajectories inferred from the transaction pattern or plain position proofs recorded in the blockchain). It also achieves high-standard security in terms of potentially malicious behavior from both the user side and the infrastructure (i.e., RSUs).

## 3. Preliminaries

### 3.1. Hash Functions

Hash functions are fundamental components in cryptography. Ideally, a hash function yields the following properties: (i) it is collision-free; that is, for a hash function H(⋅), it is infeasible to find $x_{1}$ and $x_{2}$ such that $H\left(x_{1}\right)=H\left(x_{2}\right)$; (ii) it is hiding, which means, given a hash value $y_{1}=H(x_{1})$, it is infeasible to find the corresponding $x_{1}$; and (iii) it is quick to compute the hash value H(x) for any input x. Therefore, hash functions are handy tools for verifying the integrity of messages transmitted through V2X communications. We can determine whether the message is changed by comparing the hash values of messages (usually with a small and fixed length, and thus labeled message digests) calculated before and after the transmission, no matter how large the raw message is.

### 3.2. Blockchain

**Figure 3:**
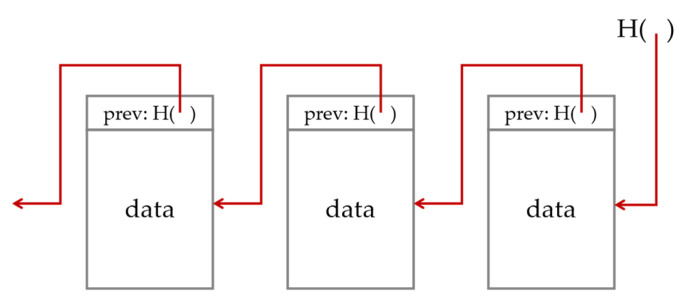
Blockchain and hash pointers (represented by the red arrows).

From the data structure perspective, a blockchain is essentially a data block list linked by hash pointers. As shown in Figure 3, each block consists of a hash pointer and block data. Unlike normal pointers, hash pointers not only consist of the storing address of the previous block but also the hash value of its content. Consequently, any changes to the data on the blockchain will result in changes to the hash pointer of the following block, which in turn will result in further changes to the blocks up until the most recent hash pointer. As the manipulation of on-chain data is easily detectable, the on-chain data are deemed immutable. In real-world applications, the data in each block are stored in Merkle trees [30] such that they can be retrieved, and this process also utilizes hash pointers to ensure data integrity.

### 3.3. Elliptic Curve Cryptography

Elliptic Curve Cryptography is a public-key cryptographic approach based on the algebraic structure of elliptic curves over finite fields [30]. Given a point P (also referred to as the generator) on an elliptic curve (EC) $y^{2}=x^{3}+ax+b$ and a secret key sk, the public key is generated using the elliptic curve scalar multiplication pk=sk⋅P, which essentially means successively adding P along the elliptic curve [30] to itself repeatedly (which implies that sk is a scalar value while pk is a point). The reliability of ECC is based on the one-wayness of the EC scalar multiplication, which means that it is infeasible to solve sk given pk.

### 3.4. Encryptions and Digital Signatures

Encryptions can be categorized into symmetric and asymmetric encryptions. In this study, we focus on public-key (asymmetric) encryption; that is, public keys are used for encryption ($e=Enc\;\left(m,\;pk\right)$, where m is the message, Enc is the encryption scheme, and e is the ciphertext), while secret keys are used for decryption: m=Dec(e, sk) [31]. In the smart vehicle context, a pair of public and secret keys can be created for each vehicle, with the public keys revealed to all as an identity and the secret keys kept by the vehicle itself. Messages sent to certain vehicles can be encrypted using their public identity/key, such that only the vehicle with the corresponding secret key can decrypt them.

Similar to public-key encryptions, digital signature schemes also use public/secret key pairs to sign signatures and verify signatures. However, in the context of digital signatures, secret keys are used for signing (sig=Sign(m, sk, r), where sig is the digital signature, Sign is the signing scheme, and r is the randomness added to the message to prevent the signature from being re-used), while public keys are used for verification, Verify(m, pk, sig) [32]. In this way, vehicles can sign on to the messages they want to disseminate with their secret keys so that anyone who received the messages can validate the authenticity and integrity of messages with the senders’ public keys.

### 3.5. MPC

In this study, MPC based on secret sharing is used. Secret sharing refers to constructing secret shares for each private input of the participants, such that each participant holds parts of secret shares, which contain no meaningful information regarding the original private inputs separately, but, together, can reconstruct the original private inputs [33]. For instance, to create secret shares for one-bit private input $x\in \left\{0,\;1\right\}$, one arbitrary bit is chosen, $x_{2}\in \{0,\;1\}$, then $x_{1}=x⊕x_{2}$ (⊕ denotes the “xor” binary operation, e.g., 0⊕0=0, 0⊕1=1, 1⊕0=1, and 1⊕1=0) and $x_{2}$ form valid secret shares of x, as no information of x is inferred with either $x_{1}$ or $x_{2}$ alone, but together, they can reconstruct x because $x=x_{1}⊕x_{2}$.

**Figure 4:**
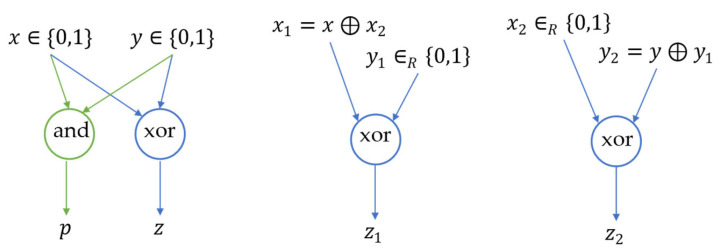
Example of secret sharing-based MPC.

Computing the outputs of a function F with private inputs using secret sharing-based MPC consists of the following steps: (i) represent F as a Boolean circuit C; (ii) generate secret shares of the private inputs of C and disseminate them to all players; (iii) evaluate C gate by gate (“gate” here refers to the Boolean gate), such that secret sharing is valid for each wire (“wires” connect the Boolean gates and transmit the outputs of upstream gates to the downstream gates as inputs); and (iv) reconstruct the function outputs on the output wires. For example, suppose two vehicles want to report to an off-ramp RSU regarding how many vehicles in total are taking the off-ramp without revealing their trips to each other using MPC. They can use a binary adder as shown in Figure 4. The Boolean circuit on the left side of Figure 4 takes two bits x and y as private indicators of whether two vehicles will take the off-ramp. The circuit consists of an “and” gate and an “xor” gate. The output consists of two bits p and z, which can form a binary representation of the total number of vehicles taking the off-ramp. Both x and y can be secret-shared, as Figure 4 shows such that vehicles 1 and 2 hold ($x_{1},\;y_{1})$ and ($x_{2},\;y_{2})$, respectively. Here, we illustrate how secret sharing remains valid on the output wire of the “xor” gate. Vehicle 1 applies the “xor” operation on $x_{1}$ and $y_{1}$ to get $z_{1}=x_{1}⊕y_{1}$ and, similarly, vehicle 2 obtains $z_{2}=x_{2}⊕y_{2}$. Since $z_{1}⊕z_{2}=\left(x_{1}⊕y_{1}\right)⊕\left(x_{2}⊕y_{2}\right)=\left(x_{1}⊕x_{2}\right)⊕\left(y_{1}⊕y_{2}\right)=x⊕y=z$; that is, we can reconstruct z with $z_{1}$ and $z_{2}$ while inferring no information about z solely with $z_{1}$ or $z_{2}$. Therefore, secret sharing holds for the “xor” gate. And secret sharing for the “and” gate also exists [34], though it is not as intuitive as that for the “xor” gate. Therefore, vehicles 1 and 2 can send all the secret shares ($p_{1},\;z_{1})$ and ($p_{2},\;z_{2)}$, respectively, to the RSU to compute the total number of vehicles taking the off-ramp.

For more complicated F, as in our proposed schemes, there are MPC compilers that can generate the corresponding circuit C, which is how we generate the MPC protocols in our numerical studies.

## 4. Privacy-Preserving Incentive Mechanism

Before presenting the details of the proposed incentive mechanism, this section starts with an introduction of the main entities: a trusted authority (TA), CVs, and RSUs, and how they are involved in the proposed incentive mechanism.

- Trusted Authority: The TA plays two essential roles in the proposed mechanism: the identity manager (I) is responsible for generating identities (public key and private key pairs) and relating vehicles’ pseudonyms to their real identities, and the mediator (M) unfreezes the frozen incentives to either refund the traffic operator when users behave dishonestly or transact the incentives to users when RSUs behave maliciously (are hacked). Therefore, TA is assumed to be fully secure and trusted. Note that though both I and M function in a centralized manner, little computation or communication burden is introduced because vehicles only request identities once with I when they participate in the mechanism for the first time. M only interacts with vehicles and RSUs under malicious behaviors, which are rare because the malicious party can be traced and penalized.
- CVs: Each CV is assumed to be able to communicate with RSUs and nearby vehicles using V2X technology. There are initiators/leaders $L^{R},\;L^{V}$, which initiate the collaborative routing scheme and the route validation scheme, respectively, and corresponding followers $F^{R},F^{V}$ The study does not consider attacks in the communication process, implying communication channels are assumed to be secure. Also, each vehicle is assumed to have a tamper-resistant device with which to store sensitive information, including a secret key and value of time securely (vehicle hardware side attacks are not considered).
- RSUs: RSUs also play multiple roles in the proposed mechanism. Some RSUs serve as checkpoint signers (S) in the route validation protocol, signing on multi-signature transactions when vehicles pass the validation at the checkpoints. And all RSUs are the nodes of the consortium blockchain, with some authorized RSUs (V) verifying the incentive transactions and position proofs and undertaking the consensus work to generate new blocks. The RSUs are semi-trusted and can be potentially malicious. However, we assume that only a small percentage of RSUs are malicious, which is widely accepted in other consortium blockchain applications [35,36].

### 4.1. Collaborative Routing Scheme

The collaborative routing scheme consists of an off-chain MPC and an on-chain incentive freezing process. The off-chain MPC takes the optimal route flows as public inputs and the value of time of vehicles in the local vehicle group as private inputs to generate route suggestions and associated incentive amounts for vehicles in the group. With the envy-freeness analysis in [21], rational users will always choose the suggested routes. After confirmation from users, the incentives will be frozen in a series of cascading multi-signature wallets for each vehicle until they pass further route validations.

First, we describe how I generates pseudonyms for vehicles that participate in the mechanism for the first time using an Elliptic Curves Cryptography (ECC) based combined-public key (CPK) scheme. Identity-based CPK derives public keys (pseudonyms) from real-world identities; hence, it does not require certificates as traditional public key encryptions, reducing the key management burden [37]. In our case, the public keys are derived from the VIN (Vehicle Identification Number) of vehicles. The identity generation process is as follows:

1. Select an elliptic curve C. Let H be an addition group of points on C, and let P be the generator of H. q∈H is an order of H (most encryption/signature schemes in this study are based on ECC; please refer to [16] for ECC basics).
2. Generate an m-length master private key vector $X=\left(x_{1},x_{2},\ldots ,\;x_{m}\right)$, where $x_{i}$ are randomly selected from $Z_{q}$.
3. Generate the corresponding public key vector $Y=\left(y_{1},y_{2},\;\ldots ,y_{n}\right)$, where $y_{i}=x_{i}\cdot P$.
4. Using X and Y, generate the private key and public key for each vehicle as follows:
  (1a) $$sk_{ID}=\sum_{i=1}^{m}h_{i}(ID)x_{i}\;mod\;q,$$
  (1b) $$pk_{ID}=\sum_{i=1}^{m}h_{i}\left(ID\right)y_{i},$$
  where $h_{i}(ID)$ is the i th bit of the digest of the vehicle’s VIN generated by $H_{0}\;:{\left\{0,\;1\right\}}^{*}\to {\left\{0,\;1\right\}}^{m}$. It is trivial to see that $pk_{ID}=sk_{ID}\cdot P$ holds, i.e., $\left(pk_{ID},\;sk_{ID}\right)$ is a valid pair of ECC keys.
5. Send vehicles their private keys through secure communication channels along with the following information as the public parameters of the cryptographic system $\left(H,q,\;Y,\left(H_{0},H_{1},H_{2},\;H_{3},\;H_{4},H_{5},\;H_{6}\right),\;E\right)$, where E is a symmetric encryption protocol (given cyphertext $y=E_{k}\;\left(x\right)$; we can obtain the plaintext $x=E_{k}^{-1}(y))$, and $H_{i}$ represents hash functions used in the schemes.

Using the pseudonyms, vehicles can initiate and participate in the collaborative routing scheme, which can be described as follows:

1. One vehicle (defined as the leader of the collaborative routing scheme $L^{R}$) initiates a request for collaborative route updating by sending nearby vehicles the message $\left\{ID_{L},\;des,\;sig_{L}\right\}$, where $ID_{L}$ is the VIN of $L^{R}$, des is the destination identifier, and $sig_{L}=sign\left(sk_{L},\;H_{1}\left(des\right)\right)$ is the signature that $L^{R}$ signs using its secret key $sk_{L}$ on the digest of des, $H_{1}\left(des\right)$.
2. Vehicles heading to the same destination and interested in joining the collaborative routing scheme (defined as the followers of the collaborative routing scheme $F^{R}$), after verifying the request ($isValid(pk_{L},\;des,\;sig_{L}$), each vehicle can reply a signed bit 1, $sig_{ID}=sign(sk_{ID},\;1)$ together with its VIN ID to $L^{R}$.
3. $L^{R}$ collects the responses from $F^{R}$, verifies the responses ($isValid\left(pk_{ID},\;1,\;sig_{ID}\right)$, and counts the total number of vehicles participating in this session, $n_{s}\;$ (which is the demand of this specific OD pair).
4. $L^{R}$ reports $n_{s}$ to the RSU nearby, which will update the flows related to this OD pair iteratively together with other RSUs in a distributed manner following the route flow assignment model in [21].
5. At the same time, $L^{R}$ and ${(n}_{s}-1)$ $F^{R}$ start establishing the communication network for the MPC protocol. $L^{R}$ produces a participation confirmation message, $\left\{<ID_{1},ID_{2},\ldots ,ID_{n_{s}}>,<sig_{ID_{1}},sig_{ID_{2}},\ldots ,sig_{ID_{n_{s}}}>\right\}$, which generates an order for all participants.
6. After $F^{R}$ receive the confirmation message, they start creating secret shares of their value of time. $\lambda_{ID_{i}}$ is denoted as the value of time of the ith vehicle ($i\in [1,\;2,\;\ldots ,\;n_{s}]$ in the confirmation message. The vehicle creates secret shares $s_{ij},\;j\in [1,\;2,\;\ldots ,\;n_{s}]$ for the jth vehicle and sends it.
7. After the RSU receives optimal route flows, it broadcasts the information as public inputs of the MPC protocol.

Note that step 6 takes the most time out of the entire process as there are $n_{s}\left(n_{s}-1\right)$ messages sent. However, this happens while RSUs are solving for the optimal flows, which is also the most time-consuming step in the hierarchical framework in [21], which mitigates the influence of step 6′s relatively long computation time.

With all required private and public inputs, the vehicles can execute the MPC protocol to produce the private outputs, which consists of their updated routes and corresponding incentives. However, MPC protocols are pre-compiled, which means that they have a fixed number of inputs, while the number of vehicles participating in the collaborative routing scheme varies in the real world. Hence, the vehicle assignment model and incentive mechanism proposed in [21] are modified as follows. Assume that the MPC protocol requires N vehicles to collaboratively update their routes (N is the maximum number of participants allowed in the scheme, determined by step 6 in practice). According to *Lemma 3* in [21], the vehicle assignment is to sort vehicles’ value of time. We can create $\left(N-n_{s}\right)$ fake vehicles with zero value of time to complement the number of inputs required by the MPC protocol. In this way, the updated routes of the participants are the same as the ones they are supposed to obtain in the vehicle route assignment model. When determining the incentives, the protocol assumes that fake vehicles take a fake route with the same travel time as the longest travel time of all real routes. According to *Lemma 4* in [21], the adjustment incentives of the fake vehicles are zero and the real vehicles’ adjustment incentives are the same as those they are supposed to obtain from the incentive mechanism in [21]. The details of the MPC protocol for route/incentive assignment (Algorithm 1) are described as follows.
**Algorithm 1.** MPC protocol for route/incentive assignment**Private input:** individual value of time $\lambda_{i},i=1,\;2,\ldots ,\;n_{s}$.

**Public input:** travel times and optimal route flows for each route $T_{k},\;f_{k},\;k\in K$, K is the route id set ($\sum f_{k}=n_{s}$).

**Public output:** incentives $p_{k}$ for each route k∈K.

Sort $\lambda_{i}$ (denote smallest as $\lambda_{min}$ and $T_{k}$ add $N-n_{s}$ vehicles with *λ_i_* = 0, and add *N* − *n_s_* flow to route with largest travel time.Duplicate *T_k_* for *f_k_* times such that there are *N* travel times in total.Assign the vehicle with the *r*th largest value of time, *λ*^(*r*)^, to the route with *r*th shortest travel time *T*^(*r*)^ (denoted as *η*^(*r*)^).$\text{Compute}\;p^{\left(1\right)}=\frac{1}{n_{s}}\sum_{j=2}^{N}\sum_{m=2}^{j}\lambda^{\left(m\right)}\left(T^{\left(m\right)}-T^{\left(m-1\right)}\right),\;and\;p^{\left(r\right)}=\;\frac{1}{n_{s}}\sum_{j=2}^{N}\sum_{m=2}^{j}\lambda^{\left(m\right)}\left(T^{\left(m\right)}-T^{\left(m-1\right)}\right)-\sum_{m=2}^{r}\lambda^{\left(m\right)}\left(T^{\left(m\right)}-T^{\left(m-1\right)}\right)$ as if there were *N* vehicles.
**Return** $\eta^{\left(r\right)}$ and $p^{\left(r\right)}$ to the vehicle with $\lambda^{\left(r\right)}.$


**Figure 5:**
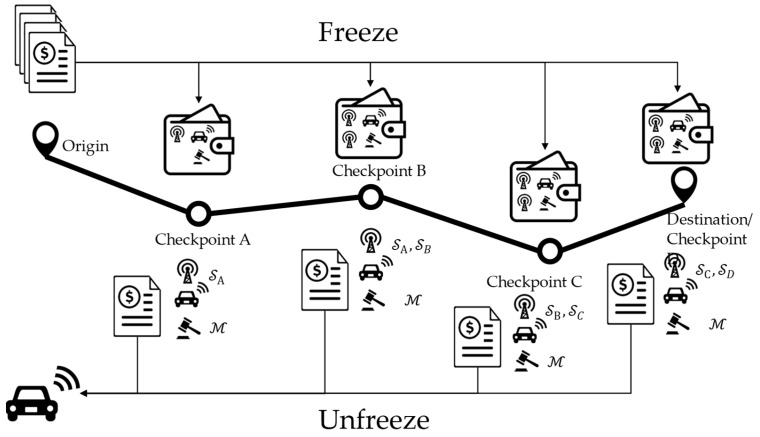
Freezing and unfreezing incentives in cascading multi-signature wallets.

The MPC protocol also generates the outputs required for the traffic manager to freeze incentives. Figure 5 shows an example of the process of freezing incentives. The route that the vehicle takes has four checkpoints A, B, C, and D. The amount of incentives that the vehicle receives for this trip is divided into four parts $a_{A},\;a_{B},\;a_{C},$ and $a_{D}$, which correspond to the four segments of the route divided by the checkpoints. To ensure that the vehicle follows the route, the traffic manager does not send the incentives to the vehicle directly at the origin. Instead, it sends the segment incentives to a series of cascading multi-signature wallets, which require multiple signatures to be authorized to transfer. The wallet corresponding to the first checkpoint is a two-out-of-three wallet, which requires at least two signatures from three wallet holders: the signer at checkpoint A, $S_{A}$, the vehicle, and the mediator M. In normal operations, $S_{A}$ signs on the transaction after the vehicle passes the route verification at checkpoint A, which makes the incentives associated with segment OA redeemable for the vehicle; it can sign on the transaction to meet the two-out-of-three signature requirement when it wants to redeem the incentives. M only plays a role when malicious behaviors are detected. Either the traffic manager or the vehicle can submit evidence to let M sign on the transaction to either refund the frozen incentives to the traffic manager or transmit the frozen incentives to the vehicle. The other wallets are three-out-of-four wallets, which require at least three signatures from four wallet holders: the signer at the upstream checkpoint, the signer at the current checkpoint, the vehicle, and the mediator.

Notably, the secret shares of the private inputs and outputs of Algorithm 1 can be fed into Algorithm 2 to skip the step of generating secret shares of the inputs of Algorithm 2. The outputs from Algorithm 2 are a series of cascading incentive freezing transactions to be signed by the traffic manager. They are marked as public because the signed transactions will be published on-chain to freeze the incentives for each segment in the corresponding multi-signature wallets. Since the signed transactions are recorded in the blockchain, the contents of $Add_{s_{j}}$ are open to everyone. However, the sensitive trip information is protected twofold. First, it is almost impossible to tell which transaction is generated for which segment incentives for whose trip, because the address of a multi-signature wallet does not explicitly show the wallet holders’ identities. As (3) shows, it is a hash of a piece of code. The public keys of the involved vehicle, checkpoints, and the mediator only appear in the code. Recall that hash functions are hiding; it is impossible to reconstruct the code using the wallet address (which is the hash of the code). It is also hard to enumerate the combinations of the wallet holders if each RSU/checkpoint has tens of valid identities registered at I. Second, each trip is divided into multiple segments, which makes identifying the entire trip of a certain vehicle exponentially harder, since it requires unhashing the wallet addresses of all involved transactions.
**Algorithm 2.** MPC protocol for incentive freezing**Private input:** individual route choices $\eta_{i}\in K,i=1,\;2,\ldots ,\;n_{s}$

**Public input:** incentives for route k denoted as $p_{k}$, lengths $l_{s_{i}},\;s_{i}\in S_{k}$ where $S_{k}$ is the segment set of route k, and $PK_{c_{j}}$, the public key set of signer $S_{c_{j}}$ at checkpoint $c_{j}\in C_{k}$ along route k∈K, where $C_{j}$ is the checkpoint set along route k.

**Public output:** transactions $TRANS_{s_{i}},\;s_{i}\in S_{k},\;k\in K$, which are to be signed by the traffic manager to freeze segment incentives.
For each individual $i=1,2,\ldots ,\;n_{s}:$ calculate
(2)$$p_{s_{j}}=\frac{l_{s_{j}}}{\sum_{s_{j}\in S_{\eta_{i}}}l_{s_{j}}}p_{\eta_{i}},\;s_{j}\in S_{\eta_{i}}.$$
To freeze segment incentive $p_{s_{j}}$, generate the following transaction $TRANS_{s_{j}}$.
** From**: the traffic manager’s address (i.e., its public key)

** To**: the address of the multi-signature wallet
(3)$$Add_{s_{j}}=H_{2}\left(script\left(pk_{i},\;pk_{M},\;pk_{r},\;pk_{m}\right)\right),\;s_{j}\in S_{\eta_{i}},$$
where $pk_{r}\in PK_{s_{j}}^{b},\;pk_{m}\in PK_{s_{j}}^{e}\;$($PK_{s_{j}}^{b}$ and $PK_{s_{j}}^{e}$ are the public key sets of the signer at the beginning and end of segment $s_{j}$, respectively), and script(⋅) is the payment script that is used to validate the transaction.

** Amount**: $p_{s_{j}}$

3.**Return** $Add_{s_{j}},\;s_{j}\in S_{\eta_{i}}$ to vehicle i.



### 4.2. Route Validation Scheme

To verify that the vehicle is at a certain checkpoint CP, $S_{CP}$ needs a position proof. Instead of using traditional GPS information (which can be easily tampered with), the position proof is generated by the witness vehicles at checkpoint CP. A reasonable assumption entails the presence of a sufficient number of vehicles in proximity to the checkpoints. Consequently, the approach delineated in this section holds significance under congested conditions, where V2X communication and privacy protection are predominantly required. To protect the privacy of the witness vehicles, threshold cryptographic techniques are used in the route validation scheme. Specifically, the signatures of real witness vehicles are mixed with other fake signatures to protect their pseudonyms from being tracked. The route validation scheme can be divided into three stages: a vehicle needing a position proof initiating a request for route validation, witness vehicles replying to the request, and verifiers validating the position proof. The first stage is as follows.

1. The vehicle that wants to prove that it is at checkpoint CP (defined as the leader of the route validation scheme $L^{V}$) generates a plaintext pos describing its current position (e.g., “on xxx link near checkpoint CP at time yyy”).
2. $L^{V}$ requests the number of witnesses required, $n_{w}$, from nearby RSUs and calculates the total number of signatures on the position proof as $n_{p}=\lceil n_{w}/\eta \rceil$, where η∈(0, 1] is the privacy protection parameter (for larger η, the pseudonyms of witness vehicles are mixed with fewer fake identities, and thus they will receive less privacy protection). That is, there should be at least $n_{w}$ nearby vehicles that witness $L^{V}\prime s$ presence at CP and they should sign on the position proof. Meanwhile, there should be another $n_{p}-n_{w}$ fake signature on the position proof as well to protect the privacy of these witness vehicles. Otherwise, anyone can learn from the position proof that these vehicles themselves are near CP at this specific time. Therefore, $L^{V}$ needs to generate and include $n_{p}-n_{w}$ fake signatures in the route validation request such, that the witness vehicles can generate signatures that cannot be distinguished from the fake signatures.
3. $L^{V}$ generates $n_{p}-n_{w}$ fake IDs from the feasible ID set, $\Omega_{f}=\{ID_{1},ID_{2},\ldots ,\;ID_{n_{p}-n_{w}}\}$ and the corresponding public keys, $pk_{ID_{j}}=\sum_{i=1}^{m}h_{i}\left(ID_{j}\right)y_{i},\;\forall j\in \{1,\;2,\;\ldots ,\;n_{p}-n_{w}\}$.
4. $L^{V}$ generates the fake signatures for the fake vehicles. For $ID_{i}\in \Omega_{f}$, it selects $a_{i},\;b_{i}\in$ $Z_{q}$ and computes:
  (4a) $$A_{i}=a_{i}\cdot P+b_{i}\cdot pk_{ID_{i}},$$
  (4b) $$\beta_{i}=-b_{i}^{-1}A_{i}[x],$$
  (4c) $$m_{i}=\alpha_{i}\beta_{i},$$
  where $A_{i}[x]$ is the x coordinate of point $A_{i}$. Note that $\left(A_{i},\;\beta_{i}\right)$ is a valid EC Elgamal signature of $m_{i}$, because $m_{i}\cdot P=A_{i}\left[x\right]\cdot pk_{ID_{i}}+\beta_{i}\cdot A_{i}$. The fake signatures are created but $L^{V}$ has no control over the corresponding $m_{i}$.
5. $L^{V}$ generates $n_{p}-n_{w}$ different indexes $\kappa_{i}=H_{3}\left(A_{i}\right)\in Z_{q},\;i\in \{1,\;2,\;\ldots ,n_{p}-n_{w}\}$ for further Lagrange polynomial interpolation.
6. $L^{V}$ initiates the request of route validation at checkpoint CP by sending the following message to the nearby vehicles: $\left\{{\left(ID_{i},\;m_{i},\;\kappa_{i}\right)}_{i=1,\;2,\;\ldots ,\;n_{p}-n_{w}},\;pos,\;n_{p},\;n_{w}\right\}$

Upon receiving the route validation request, nearby vehicles that identify $L^{V}$ at checkpoint CP will reply to the request with a signed message back, becoming witness vehicles in $L^{V}$’s position proof. A witness vehicle $F^{V}$ generates its signature as follows:

1. $F^{V}$ constructs a polynomial f with $n_{p}-n_{w}$ degrees defined on Galois field $GF(2^{n_{l}})$($A_{i}\left[x\right]\in GF\left(2^{n_{l}}\right)$) using Lagrange interpolation, such that $f\left(0\right)=H_{4}\left(des\right)$ and $f\left(\kappa_{i}\right)=E_{k}\left(m_{i}\right),\;i=1,\;2,\;\ldots ,\;n_{p}-n_{w}$, where $k=H_{5}(n_{p}||n_{w})$.
2. $F^{V}$ chooses a random index $\kappa \notin \left\{\kappa_{1},\;\kappa_{2},\ldots ,\;\kappa_{n_{p}-n_{w}}\right\}$ and generates $m=E_{k}^{-1}\left(f\left(\kappa \right)\right).$
3. $F^{v}$ randomly selects $c\in Z_{q}$ and generates the EC Elgamal signature (A, β) of m, where $A=c\cdot P,\;\beta =\left(m-sk\cdot A[x]\right)c^{-1}.$
4. $F^{v}$ replies to the route validation request by sending a message, {ID, m, κ, (A, β)}, to $L^{V}$.

Once $L^{V}$ receives more than $n_{w}$ responses from the nearby vehicles, it aggregates the fake and collected signatures to generate a position proof P and sends it to the verifiers V:

$$P=\left\{{\left(ID_{i},\;m_{i},\;\kappa_{i},\;(A_{i},\;\beta_{i})\right)}_{i=1,\;2,\;\ldots ,\;n_{p}},\;pos,\;n_{p},n_{w}\right\}.$$

Each V then verifies the position proof P as follows:

1. V generates the public keys $pk_{ID_{j}}=\sum_{i=1}^{m}h_{i}\left(ID_{j}\right)y_{i},\forall j\in \{1,2,\ldots ,n_{p}\}$.
2. V verifies whether the signatures $\left(A_{i},\beta_{i}\right)$ are valid by checking whether $m_{i}\cdot P=A_{i}\left[x\right]\cdot pk_{ID_{i}}+\beta_{i}\cdot A_{i}$ holds for $i\in \left\{1,2,\ldots ,n_{p}\right\}$.
3. V randomly selects $n_{p}-n_{w}$ tuples from ${\left(ID_{i},m_{i},\kappa_{i},\left(A_{i},\beta_{i}\right)\right)}_{i=1,2,\ldots ,n_{p}}$, reconstructs the polynomial f using Lagrange interpolation, such that $f\left(0\right)=H_{4}\left(des\right)$ and $f\left(\kappa_{j}\right)=E_{k}\left(m_{j}\right)$, where $k=H_{5}(n_{p}||n_{w})$, and verifies whether $f\left(k_{i}\right)=E_{k}(m_{i})$ holds for all i≠j.
4. V accepts the position proof if it passes all verifications.

To prevent malicious behavior by a single V, the position proof is sent to all V in the RSU network, and the PBFT algorithm [38] is used to generate a consensus mechanism. The presence of malicious nodes will not impact the final consensus when the number of malicious nodes is less than one-third of the participating nodes. When consensus is achieved that the position proof is valid, the digest of the position proof, $H_{6}\left(P\right)$, instead of the plaintext P, is written to the latest block of the blockchain. $S_{CP}$ will sign on the unfreezing transaction after verifying that the position is in the blockchain (which indicates that the position proof has been verified by the majority of the verifiers).

### 4.3. Privacy and Security Analysis

To ensure that the proposed schemes can prevent privacy leaks and ensure consistency between the routes that vehicles choose and the ones they take, this section analyzes the potential privacy leaks in the scheme steps and discusses how malicious behavior can be managed in the incentive mechanism.

First, the proposed schemes are privacy-preserving in both the collaborative routing and the route validation processes. In the collaborative routing scheme, $L^{R}$ and $F^{R}$ hide their real identities with pseudonyms when sending messages. Vehicles receiving messages only know that some vehicles are heading to des but cannot connect these messages to nearby vehicles. Also, as the messages are kept off-chain, it is hard to connect the pseudonyms to real identities through pattern analysis. The messages sent to RSUs are aggregated information, $n_{s}$. Therefore, no individual privacy information is leaked when RSUs compute optimal route flows. When computing updated routes and incentives thatfollow the MPC protocol, vehicles only send secret shares of their value of time to other vehicles, and outputs are generated by each vehicle using these shares. No meaningful information can be inferred from incomplete shares. In the on-chain incentive freezing process, incentives are sent to different multi-signature wallets. Note that S of multi-signature wallets do not need to be the RSUs near the corresponding checkpoints (because verifying position proofs is a cryptographic process independent of the RSU position). Therefore, wallet addresses only provide random signers’ public keys, which cannot be used for privacy pattern analysis. In route validation schemes, the pseudonyms of $L^{V}$ and $F^{V}$ are mixed with fake pseudonyms in position proofs, which provides additional privacy protections, as position proofs are sent to all RSUs for PBFT consensus. Before logging into the blockchain, the position proofs are hashed to ensure no position/route information can be inferred from pattern analysis of the information in the blockchain.

Behavioral honesty can also be illustrated for both the collaborative routing and the route validation processes. Given that the MPC protocol ensures no privacy leakage risks, vehicles participating in collaborative routing have no privacy concerns. And [21] has shown that under this condition, vehicles will behave honestly (i.e., provide genuine inputs to the collaborative routing scheme) to maximize their own utilities. In the route validation scheme, first, the cryptographic tools used in the scheme design mitigate common types of attacks. The identity-based asymmetric key generation mitigates Sybil attacks, in which a single entity operates multiple fake identities simultaneously to undermine the system by gaining the most influence in the network. Also, the EC digital signature algorithm widely applied in the proposed scheme ensures that signatures cannot be forged, and messages are tamper-resistant. Therefore, adversaries cannot launch replay attacks in the route validation scheme by re-sending messages they received before. Also, $L^{V}$ cannot generate a fake position proof by forging more than $n_{p}-n_{w}$ signatures. Because $L^{V}$ has no control over the $m_{i}$ corresponding to fake signatures, it can forge at most $n_{p}-n_{w}$ signatures to identify $n_{p}-n_{w}$ points on $GF\left(2^{l}\right)$ (there is an additional point $\left(0,\;H_{4}\left(des\right)\right)$) and determine a polynomial with degree $n_{p}-n_{w}$. If it forges more signatures, it cannot ensure that the corresponding $m_{i}$ is on the polynomial, which can be easily detected by V.

## 5. Numerical Studies

Simulation studies are conducted to illustrate the performance of the privacy-preserving incentive mechanism. First, we show the correctness of the proposed incentive mechanism. Then, the computational efficiency of the collaborative routing scheme and the route validation scheme is analyzed under different privacy protection settings. The MPC protocols are implemented in SCALE-MAMBA (https://github.com/KULeuven-COSIC/SCALE-MAMBA (accessed on 9 January 2024)) and MP-SPDZ (https://github.com/data61/MP-SPDZ (accessed on 9 January 2024)), and the route validation scheme is implemented using Python.

**Figure 6:**
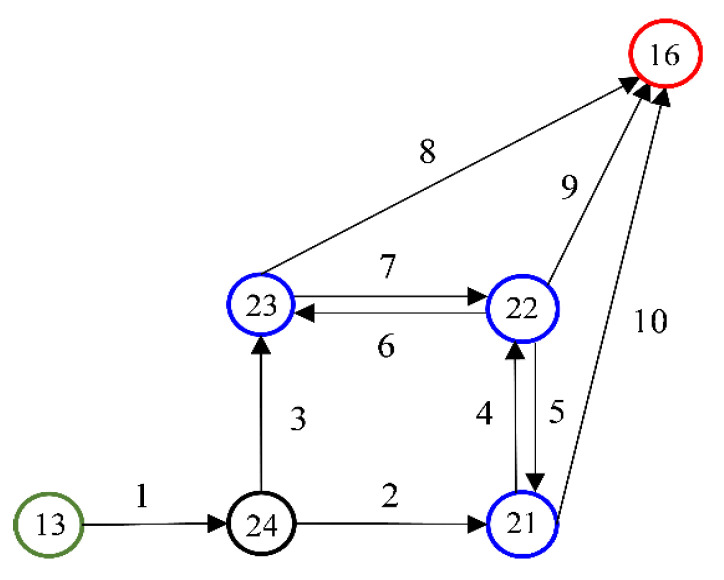
Example local road map from [21] (the blue circles are local destinations; the green and red circles are the origin and final destination; and the link/node IDs are denoted in the figure).

To validate the correctness of the MPC protocol, the example network (see Figure 6) in [21] is used to illustrate that the MPC protocol can calculate the same incentives with proper settings. Twenty vehicles depart from node 13 to node 16 in the network. There are three local destinations, nodes 21, 22, and 23, and four alternative routes connecting nodes 13 and 16, as listed in Table 1 and illustrated in Figure 6. The desired flows and the corresponding travel costs of the four routes are provided by the route flow assignment model in [21]. The values of time () for the individual vehicles are shown in Table 2. With these inputs, the implemented MPC scheme can generate the same vehicle route assignment results $\eta_{i},\;i=1,\;\ldots ,\;20$ (the id of the route that vehicle i should take) as in [21]. In terms of the incentives, when the integer representation precision of the MPC protocol is set as 15-bit fixed point numbers with a 5-bit decimal part, the output incentives $p_{i}^{\left(5,\;15\right)}$ are different from the ones calculated in [21] (although $\sum_{i=1}^{20}p_{i}^{\left(5,\;15\right)}=0$ holds as $\sum_{i=1}^{20}p_{i}=0$). If the precision is increased to 31-bit fixed point numbers with a 16-bit decimal part, the output incentives $p_{i}^{\left(16,\;31\right)}=p_{i},\;i=1,\;\ldots ,\;20$ (i.e., the implemented MPC scheme can calculate the same incentives as in [21] under this setting). The total data exchanged among the vehicles when executing the MPC scheme increase from 135.478 MB to 180.649 MB in this case, which is acceptable in the real world.

**Figure 7:**
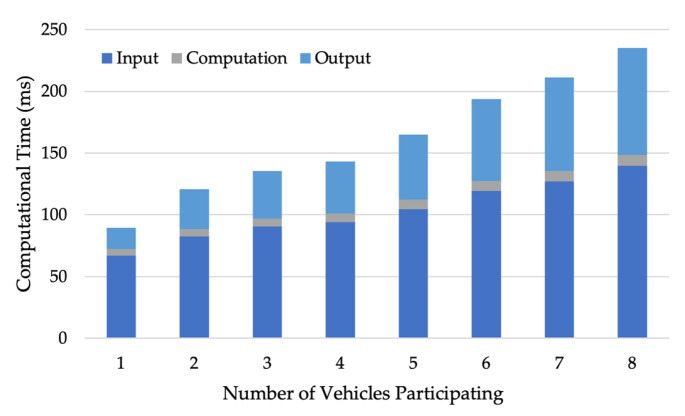
Computational times of the collaborative routing scheme.

Next, we validate the computational efficiency of the collaborative routing scheme. Due to our modifications (fake vehicles and routes) to the incentive mechanism, the compiled MPC protocol can be executed by fewer vehicles than the predefined number of parties, N. Figure 7 shows how the computational time of different stages changes with the number of participating vehicles ($n_{s})$ given a compiled 8-party MPC protocol (N=8). The computational times of the input and output stages increase with $n_{s}$ because fake vehicles’ inputs are pre-determined and do not require outputs. The computation time for the computation stage slightly increases as the number of fake vehicles decreases.

**Figure 8:**
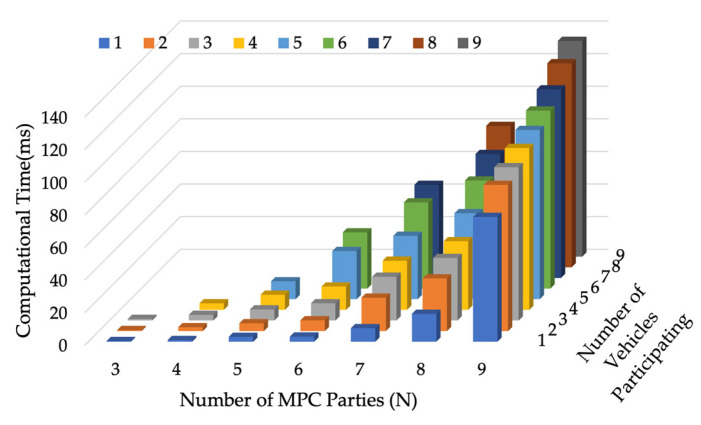
MPC output generation times under different N and $n_{s}$.

Figure 8 shows how the computational time of the output stage changes with N and $n_{s}$ ($n_{s}\leq N)$. It increases significantly with an increase in the number of MPC parties. To enable real-time implementation, the vehicle group size should be limited to 10. When more than 10 vehicles want to participate, they can be assigned into multiple vehicle groups with the same OD. The flow updating model can accommodate such settings. Also, the simulations were conducted on one desktop with one thread, while in real implementation, outputs can be generated parallelly on all participating vehicles, which can reduce the total computational time as well. Given the exponential increase in the MPC scheme computational time with the increase in MPC parties, a potential refinement is to subdivide the collaborative routing problem into smaller portions. This process would enable the MPC schemes to be executed with a reduced number of parties involved in each smaller subproblem. The results obtained from these smaller subproblems can then be aggregated by another MPC scheme. Structuring MPC in this hierarchical way is likely to address the computational issue associated with a large number of MPC parties.

**Figure 9:**
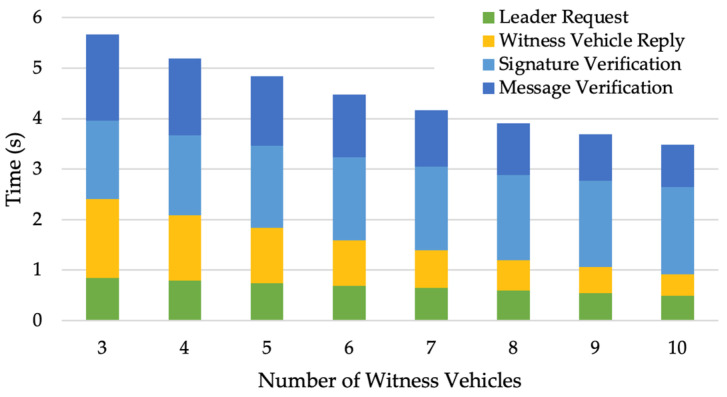
Computational times of the route validation scheme with different numbers of witness vehicles when $n_{p}=20$.

Next, the computational feasibility of the route validation scheme is evaluated. The results in Figure 9 seem counter-intuitive at first glance; the time the leader vehicle takes to generate the request, the time that witness vehicles take to reply to the leader, and the total computational time all decrease as the number of witness vehicles increases. Since $n_{p}$ is fixed, an increase in the number of witness vehicles will result in a decrease in the number of fake signatures that the leader generates; thus, the leader request time is reduced. Also, when witness vehicles generate the replies, the most time-consuming step is constructing the polynomial based on all fake signatures, which takes more time as the number of fake signatures increases. Therefore, the reply times of witness vehicles indicated by the yellow bars in Figure 9 decrease as the number of witness vehicles increases.

**Figure 10:**
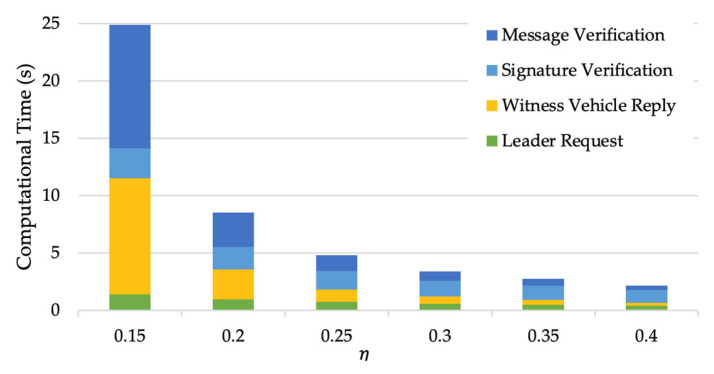
Computational times of the route validation scheme under different values of privacy protection parameter η.

To evaluate how the value of the privacy protection parameter η influences the efficiency of the route validation scheme, we compare the computational time of simulations with different η  when the number of required witness vehicles $n_{w}=5$. Figure 10 shows that the computational time decreases significantly as η increases, especially the time that witness vehicles take to generate a reply message and the message verification time of V.

It should be noted that the collaborative routing scheme and route validation scheme encompass a variety of critical cryptographic operations, including the EC Elgamal key generation algorithm and signature algorithm. The time complexity of these components is substantially influenced by the parameters of the cryptographic settings, such as the order q of the addition group H. This section predominantly focused on analyzing the effects of parameters that hold greater relevance to the transportation context. In practical applications, the selection of these parameters must strike a balance between privacy security and computational efficiency. Securing privacy is certainly a crucial aspect, but the emphasis should equally be on computational feasibility, especially for vehicular applications. Generally, enhancing privacy protection could imply a potential trade-off in computational efficiency. For instance, using a smaller η affords better privacy protection for witness vehicles, as more fake signatures are blended into the position proof. However, this could concurrently increase the computational times, as indicated in Figure 10, thus impacting the route validation scheme’s efficiency. Hence, a careful trade-off must be enabled in practice.

## 6. Conclusions

This study offers several significant contributions. First, collaborative routing facilitates personalization by accounting for user heterogeneities, leading to increased privacy concerns. To address this issue, the proposed method combines MPC with collaborative routing, thereby enabling privacy-preserving collaboration and showcasing a potential solution to privacy concerns associated with personalization. Second, the study introduces a V2V-based position proof approach as an alternative to the widely used GPS, which has raised concerns regarding the sharing of privacy-sensitive information. This alternative allows users to verify their travel history without disclosing their historical positions, a characteristic that has not been achieved previously. Third, the study presents a novel on-chain/off-chain structure that capitalizes on the tamper-resistance property of on-chain data while maintaining sensitive privacy pattern information off-chain. This design offers valuable insights into harnessing the benefits of blockchain technology while circumventing privacy risks associated with its inherent transparency. It should be emphasized that the suggested approach extends significantly beyond the scope of [21], as the primary objective of the current study is to address potential privacy breaches arising from personalization within transportation systems. The MPC framework may be adapted to alternative application contexts involving personalized demand-side solutions. Furthermore, the position verification technique can be employed in additional applications necessitating the sharing of travel history, thereby safeguarding user privacy.

Potential directions for future research include: (i) making position proofs reusable for witness vehicles to reduce duplication of verification; (ii) refining the MPC structure to allow more vehicles in one vehicle group; and (iii) incorporating the incentive mechanism into the broader intelligent transportation system to form a sustainable incentive ecosystem, where users can spend the incentives they gain, such that pseudonyms do not need to be connected to bank accounts, further protecting privacy.

## Figures and Tables

**Table 1:** Alternative routes with desired route flows and travel costs.

Route ID	Links	Flow	Travel Cost
1	1-2-10	8	289.961
2	1-2-4-9	2	290.086
3	1-3-7-9	1	331.512
4	1-3-8	9	331.500

**Table 2:** Vehicle incentives comparison.

Vehicle ID	$\lambda_{i}$	$\eta_{i}$	$a_{i}$	$p_{i}$	$p_{i}^{\left(5,15\right)}$	$p_{i}^{\left(16,31\right)}$
1	0.80	1	0.000	−12.401	−12.375	−12.401
2	0.91	1	0.000	−12.401	−12.375	−12.401
3	0.45	4	24.786	12.385	12.313	12.385
4	0.46	4	24.786	12.385	12.313	12.385
5	0.72	1	0.000	−12.401	−12.375	−12.401
6	0.64	2	0.080	−12.321	−12.281	−12.321
7	0.54	4	24.786	12.385	12.313	12.385
8	0.84	1	0.000	−12.401	−12.375	−12.401
9	0.61	2	0.080	−12.321	−12.281	−12.321
10	0.42	4	24.786	12.385	12.313	12.385
11	0.60	4	24.786	12.385	12.313	12.385
12	1.00	1	0.000	−12.401	−12.375	−12.401
13	0.40	4	24.786	12.385	12.313	12.385
14	0.43	4	24.786	12.385	12.313	12.385
15	0.87	1	0.000	−12.401	−12.375	−12.401
16	0.76	1	0.000	−12.401	−12.375	−12.401
17	0.23	4	24.786	12.385	12.313	12.385
18	0.71	1	0.000	−12.401	−12.375	−12.401
19	0.49	4	24.786	12.385	12.313	12.385
20	0.15	3	24.788	12.387	12.313	12.387

## Data Availability

Data are contained within the article.
